# Time-resolved RNA signatures of CD4+ T cells in Parkinson’s disease

**DOI:** 10.1038/s41420-023-01333-0

**Published:** 2023-01-21

**Authors:** Caroline Diener, Martin Hart, Tim Kehl, Anouck Becker-Dorison, Tanja Tänzer, David Schub, Lena Krammes, Martina Sester, Andreas Keller, Marcus Unger, Barbara Walch-Rückheim, Hans-Peter Lenhof, Eckart Meese

**Affiliations:** 1 Institute of Human Genetics, Saarland University, 66421 Homburg, Germany; 2 Center for Bioinformatics, Saarland Informatics Campus, Saarland University, 66123 Saarbrücken, Germany; 3 Department of Neurology, University Hospital of Saarland, 66421 Homburg, Germany; 4 Institute of Virology and Center of Human and Molecular Biology, Saarland University, 66421 Homburg, Germany; 5 Department of Transplant and Infection Immunology, Saarland University, 66421 Homburg, Germany; 6 Chair for Clinical Bioinformatics, Saarland University, 66123 Saarbrücken, Germany; 7 Helmholtz-Institute for Pharmaceutical Research Saarland (HIPS), Helmholtz-Centre for Infection Research (HZI), 66123 Saarbrücken, Germany; 8 Department of Neurology, SHG Sonnenberg, 66119 Saarbrücken, Germany

**Keywords:** Immunology, Mechanisms of disease, Parkinson's disease

## Abstract

Parkinson’s disease (PD) emerges as a complex, multifactorial disease. While there is increasing evidence that dysregulated T cells play a central role in PD pathogenesis, elucidation of the pathomechanical changes in related signaling is still in its beginnings. We employed time-resolved RNA expression upon the activation of peripheral CD4+ T cells to track and functionally relate changes on cellular signaling in representative cases of patients at different stages of PD. While only few miRNAs showed time-course related expression changes in PD, we identified groups of genes with significantly altered expression for each different time window. Towards a further understanding of the functional consequences, we highlighted pathways with decreased or increased activity in PD, including the most prominent altered IL-17 pathway. Flow cytometric analyses showed not only an increased prevalence of Th17 cells but also a specific subtype of IL-17 producing γδ-T cells, indicating a previously unknown role in PD pathogenesis.

## Introduction

Parkinson’s disease (PD) is one of the most common neurodegenerative disorders with a still increasing worldwide incidence [1–3]. Cardinal PD motor symptoms include tremor, bradykinesia, rigidity and postural instability, which are connected to the loss of dopaminergic neurons of the *substantia nigra pars compacta* [4]. The pathological formation of α-synuclein aggregates and Lewy bodies has been associated with neuronal degeneration [4–6].

However, it is becoming increasingly evident that factors outside the brain are also significant contributors to PD pathogenesis. Deregulated immune cells could play a central role in the according scenarios [7–9]. Especially CD4+ T cells are of major importance for a coordinated immune function and appear to be deregulated in context of PD as they invade the brain and likely induce an autoimmune reaction [10–12]. Since functional T cell deregulation seems to emerge long time before the manifestation of PD cardinal motor symptoms, T cell changes also bear a great potential of being utilized as biomarkers for early diagnostics [12, 13]. Additionally, T cell changes offer themselves as a starting point for the development of novel (immune) therapeutic strategies [8, 14]. However, the pathomechanistic deregulation of T cell pathways awaits further clarification [8, 15, 16].

Time-course RNA expression data are particularly suitable to track and functionally relate changes on cellular signaling pathways [17, 18]. Thus, to decipher deregulated T cell signaling in context with PD, we analyzed time-resolved RNA expression dynamics upon the activation of peripheral CD4+ T cells.

Based on the examination of five representative PD cases at different stages of disease, our study highlights comprehensive RNA expressional deregulations and identifies pathways related to changes on T cell functionalities in PD. Our data point to a mitochondrial dysfunction and a deregulation of DNA repair and are indicative for an increase of various autoimmune features and also provide first evidence for a yet unknown role of IL-17 producing gamma delta T cells in PD pathogenesis.

## Results

### Experimental setup and quality control

To uncover deregulated T cell signaling in Parkinson’s disease (PD), we analyzed peripheral CD4+ T cells from five PD patients in an age range between 53–86 and at different stages of disease i.e., stage 4, 4, 2.5, 1, and 1 according to the Hoehn & Yahr scale. Patient 5 was analyzed immediately upon diagnosis. As control we used five blood controls (healthy controls (HCs)) from healthy donors, aged 53–69.

**Fig. 1:**
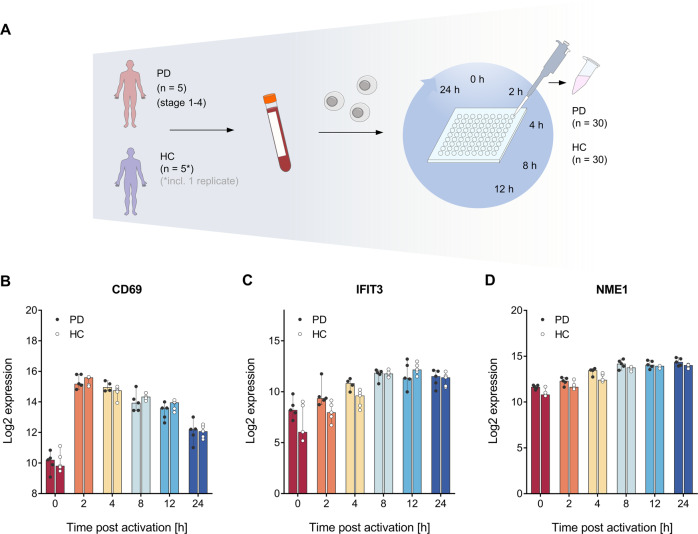
Overview on the experimental setup and comparative analysis of T cell activation markers. **A** PD patients at different disease stages and gender matched healthy controls (HCs) of similar age were recruited. Peripheral blood samples were collected and CD4+ T cells were isolated by negative selection. T cell activation was in vitro stimulated and cell samples were collected at 0, 2, 4, 8, 12, and 24 h resulting in of 60 time-course samples (PD (*n* = 30); HC (*n* = 30)). **B**–**D** Effective induction of the T cell activation process for both PD patients and HCs was confirmed for the different time-points by using the T cell activation markers CD69 (**B**), IFIT3 (**C**), and NME1 (**D**). A log2 mRNA expression scale is shown on the y-axis. Median results are indicated by bars, total expression ranges per group are illustrated by single data points.

Since the initial 24 h time frame of the activation is decisive for a proper T cell immune function, including shifts of central signaling pathways and the transition from a resting to a proliferative cell stage [17, 19, 20], we induced the activation of the isolated CD4+ T cells by in vitro stimulation. Time-course sampling was done at different time-points (0, 2, 4, 8, 12, and 24 h), resulting in a total of 60 RNA samples (PD (*n* = 30); HC (*n* = 30)). These RNA samples were used for high-throughput analysis of time-resolved RNA expression profiles. An overview of the experimental setup is given in Fig. 1A. RNA integrity number (RIN) values of the extracted total RNA ranged from 7.1 to 9.1 indicating a high RNA quality for all time-course samples. Since CD69, IFIT3, and NME1 are reliable markers for the early 24 h T cell activation phase [21], we used them to verify the effective induction of the T cell activation process. Comparability between the two groups (PD patients and HCs) was confirmed by overlapping CD69, IFIT3, and NME1 mRNA expression patterns (Fig. 1B–D).

### Detection of differential transcriptomics during the time-course of T cell activation

Analyses on the time-course transcriptional data by data clustering and multidimensional scaling identified distinct expressional changes between the PD and HC group. In detail, we calculated the pairwise Euclidean distance between the samples, across all genes and across all the time-points. We then applied the cmdscale function of the R base library to perform a classical multidimensional scaling with two dimensions (Parameters: eig = TRUE, *k* = 2). The combined time-course data allowed a clear separation between PD patients and HCs (Supplementary Fig. 1A, horizontal axis). While patients at an early stage of PD (P4 and P5) mapped closer to HCs, patients with a progressive stage mapped at a greater distance to HCs, indicating of a possible link between clustering and severity or duration of PD. However, due to the small number of patients and controls, our study focuses on the separation between controls and PD patients considering both, early and late stages of the Parkinson´s disease.

**Fig. 2:**
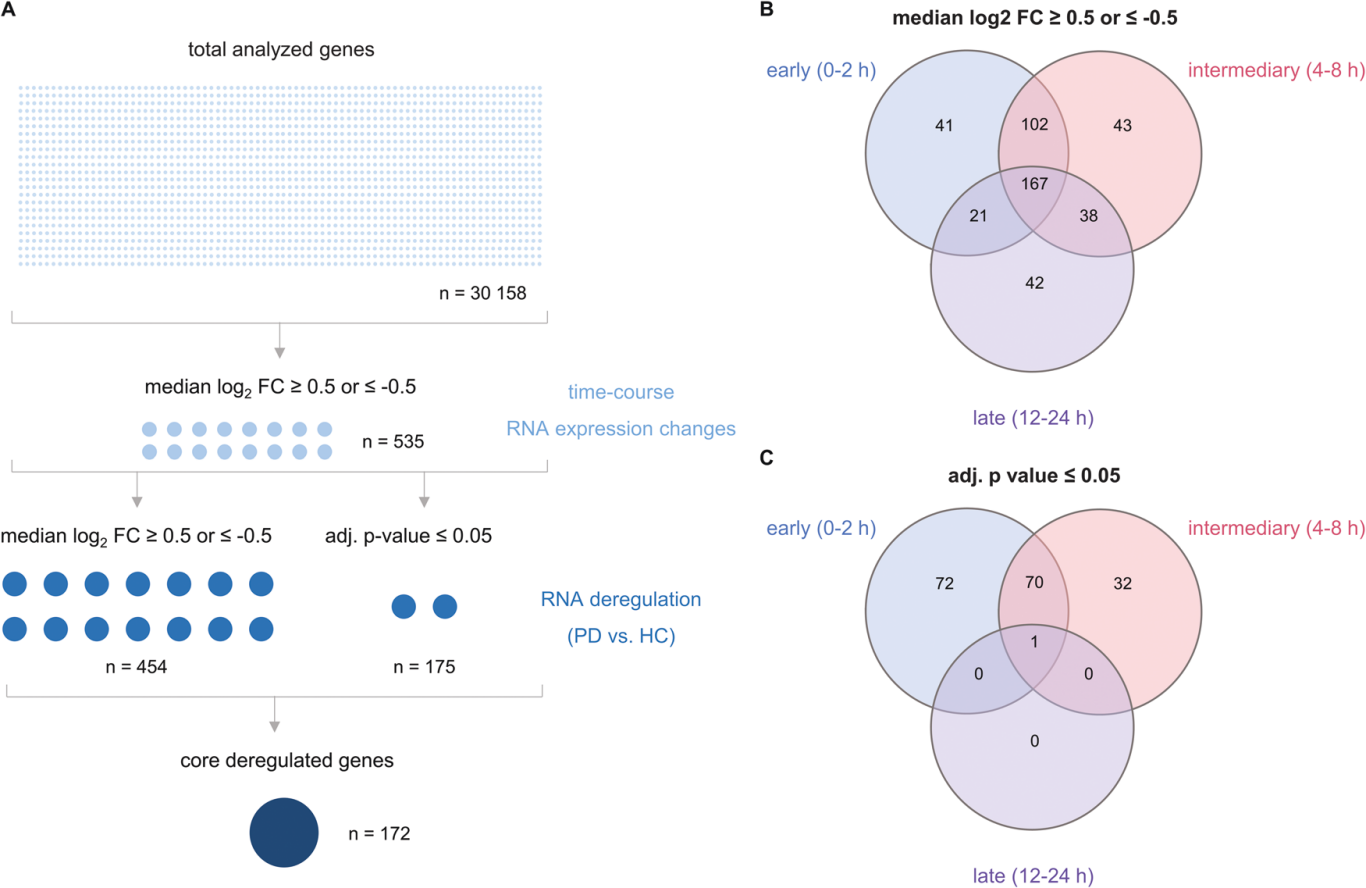
Determination of differential gene expression in context with T cell activation and numerical comparison of deregulated genes. **A** Following T cell activation a total of 30,158 genes was analyzed for time-course RNA expression changes. There were 535 genes with a median log_2_ fold change (FC) ≥0.5 or ≤−0.5 during the time-course. The comparison between the PD and HC groups showed 454 genes with a median log_2_ FC ≥0.5 or ≤−0.5 and 175 genes with a significantly altered expression (*p*-values ≤ 0.05 adjusted by Benjamini & Hochberg). The 172 genes that showed both a significantly altered expression and a median log_2_ FC ≥0.5 or ≤−0.5 are referred to as core deregulated genes. **B**, **C** The Venn diagrams provide a numerical summary of genes that were deregulated at different time windows upon T cell activation. The diagram (**B**) includes the genes with a median log_2_ FC ≥0.5 or ≤−0.5 and the diagram (**C**) the genes with an adjusted *p*-values ≤ 0.05.

A total of *n* = 30 158 genes were analyzed by our time-resolved RNA expression analyses. We found 535 genes with a median log_2_ fold change of ≥0.5 or ≤−0.5 during the time-course. Out of the resulting 535 genes, 454 genes additionally showed a median log_2_ fold change of ≥0.5 or ≤−0.5 for the comparison between PD patients and HCs. For the same comparison we found 175 deregulated genes with significantly different expression (*p*-values adjusted by Benjamini & Hochberg (adj. *p*-value ≤ 0.05) (Fig. 2A).

We next addressed the question how many genes were differentially expressed in different time windows between PD patients and HCs. While expression changes were found throughout the entire time course, the significant changes were mainly attributed to the early and intermediary phases as detailed in the following. Likewise, multidimensional scaling under itemization of the different time-points, specified a clear distinction between PD patients and HCs, particularly for the early hours (0–4 h) of the T cell activation time-course (Supplementary Fig. 1B).

Out of the 454 genes differentially expressed between PD and HCs with a median log_2_ fold change of ≥0.5 or ≤ −0.5, there were 41 genes exclusively within the early time window between 0–2 h, 43 genes exclusively within the intermediary phase between 4–8 h, and 42 genes exclusively in the late phase between 12–24 h. There was an overlap of 102 genes that were shared between the early and intermediary phases, 38 genes shared between the intermediary and late phases and 167 genes that were found throughout all phases of the T cell activation course (Fig. 2B).

Out of the 175 genes with a significantly altered expression, there were 72 genes specifically for the early time window and 32 genes specifically for the intermediary time window. There were no genes shared between the intermediary and late phases. Likewise, no genes were shared between the early and late phases. A single gene (*HCAR3*) was found in all phases of the T cell activation course (Fig. 2C). As exemplified for *VCAN1* in the sections below, not all of the genes showed uniform directions of deregulations throughout the entire time-course.

**Fig. 3:**
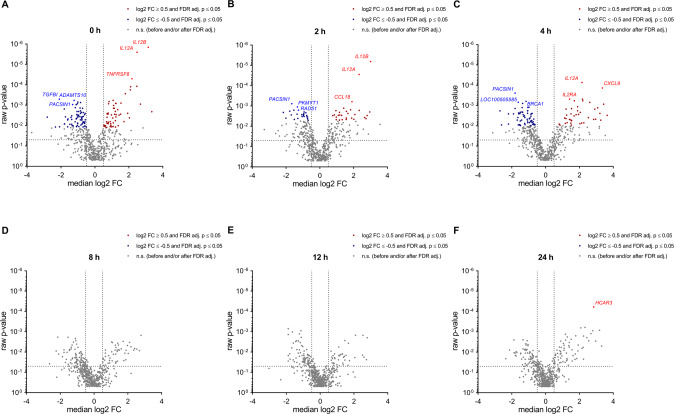
Time-course analyses reveal differential gene expression in context with T cell activation. The volcano plots show RNA expressional changes between PD and HC for each of the analyzed time-points after T cell activation (0, 2, 4, 8, 12, 24 h; **A**–**F**). Core deregulated genes with an adjusted *p*-value ≤ 0.05 and a median log_2_ FC ≥0.5 or ≤−0.5 are highlighted in red for an increased expression in PD samples and in blue for a decreased expression in PD samples. Examples of top deregulated genes are denoted and indicated by a slightly lighter color.

Overall, we identified 172 core deregulated genes with both, a significantly altered expression (adj. *p*-value ≤ 0.05) and a median log_2_ fold change of ≥0.5 or ≤ −0.5 for the comparison between PD samples and HCs (Fig. 2A). In the following we address the different time points and provide examples of top deregulated genes (Fig. 3). Out of the core deregulated genes there were 117 genes with an increased RNA expression in PD at the 0 h time-point. Some of the most extensively increased genes at this time-point were *IL12B*, *IL12A* and *TNFRSF8. TGFBI*, *ADAMTS10* and *PACSIN1* were amongst the top genes with a decreased RNA expression in PD. At time points 2 h and 4 h, there were 53 and 117 of the core deregulated genes, respectively. Among the most extensively increased genes were *IL12B*, *IL12A* and *CCL18* at the 2 h time-point and *IL12A*, *CXCL9* and *IL2RA* at the 4 h time-point. Examples for the top genes with a decreased RNA expression were *PACSIN1*, *PKMYT1* and *RAD51* at the 2 h and *PACSIN1*, *LOC100505585* and *BRCA1* at the 4 h time-point, respectively. As for the later time points there were no deregulated genes with a significantly altered expression and a median log_2_ fold change of ≥0.5 or ≤−0.5 at the 8 h and 12 h. However, at the 24 h time-point *HCAR3* showed an increased expression meeting these criteria.

### Specification of deregulated T cell gene expression signatures in context with PD

In the following, we focus on those core deregulated genes (i.e., genes with an adj. p-value ≤0.05 and median log_2_ fold change of ≥0.5 or ≤−0.5 between PD and HC) that also show a significant deregulation at more than one time-point.

**Fig. 4:**
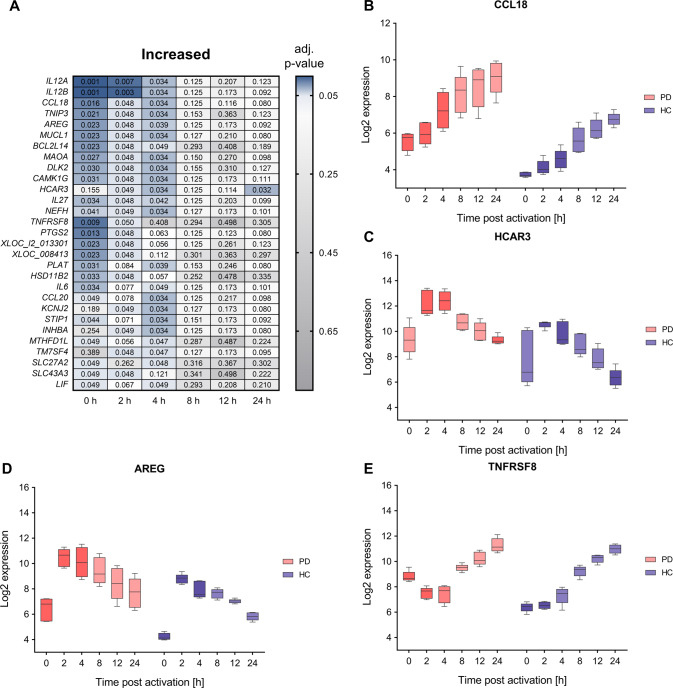
Comparative analysis of time-course transcriptomics data show increases of T cell gene expression in PD. **A** A total of 29 core deregulated genes with an adjusted *p*-value ≤ 0.05 and a median log_2_ FC ≥0.5 or ≤−0.5 for the comparison between PD and HC groups showed a significant RNA increase at more than one time-point of the T cell activation course. Corresponding FDR adjusted *p*-values of the analyzed time-points (0, 2, 4, 8, 12, and 24 h) are depicted for the comparison between PD and HC groups. **B**–**E** Exemplary log2 time-course expression data of CCL18, AREG, HCAR3, and TNFRSF8 mRNAs (**B**, **C**, **D**, **E**, respectively) are shown for PD and HC groups. The boxes show the range from the 25th to 75th percentiles with the median results indicated by horizontal lines within the boxes and the total expression ranges by whiskers. Significant differences between PD and HC at the time-points are indicated by darker colors.

In total we identified 29 core deregulated genes with significantly increased RNA expression at least at two time-points during the overall T cell activation time-course (Fig. 4A). Corresponding genes encode among others the chemokine ligand CCL18, the growth factor and type 2 cytokine AREG, the mitochondrial monoamine oxidase MAOA, the hydroxycarboxylic acid receptor HCAR3 and the TNF receptor TNFRSF8 (Fig. 4B–E). CCL18 mRNA showed significant expressional deregulation between PD and HC samples during 0–4 h. Median log_2_ fold changes for the comparison between PD and HC samples within this timeframe were in the range 1.90 to 2.61. Corresponding FDR adjusted *p*-values were in the range of 4.79 × 10^−2^–1.57 × 10^−2^. Likewise, AREG and MAOA mRNAs showed significant expressional deregulation between PD and HC samples during 0–4 h. Median log_2_ fold changes were in the range of 1.82 to 2.68 for AREG and of 1.09 to 1.57 for MAOA. Corresponding FDR adjusted *p*-values ranged from 2.29 × 10^−2^–4.79 × 10^−2^ and 2.70 × 10^−2^–4.79 × 10^−2^, respectively. The HCAR3 mRNA showed significant deregulation during 2–4 h and at the 24 h time-point. Corresponding median log_2_ fold changes ranged from 0.97 to 3.08 and FDR adjusted p-values ranged from 3.17 × 10^−2^–4.91 × 10^−2^. The TNFRSF8 mRNA showed significant deregulation during 0–2 h. Corresponding median log_2_ fold changes ranged from 1.16 to 2.18 and FDR adjusted p-values ranged from 8.97 × 10^−3^–4.95 × 10^−2^.

**Fig. 5:**
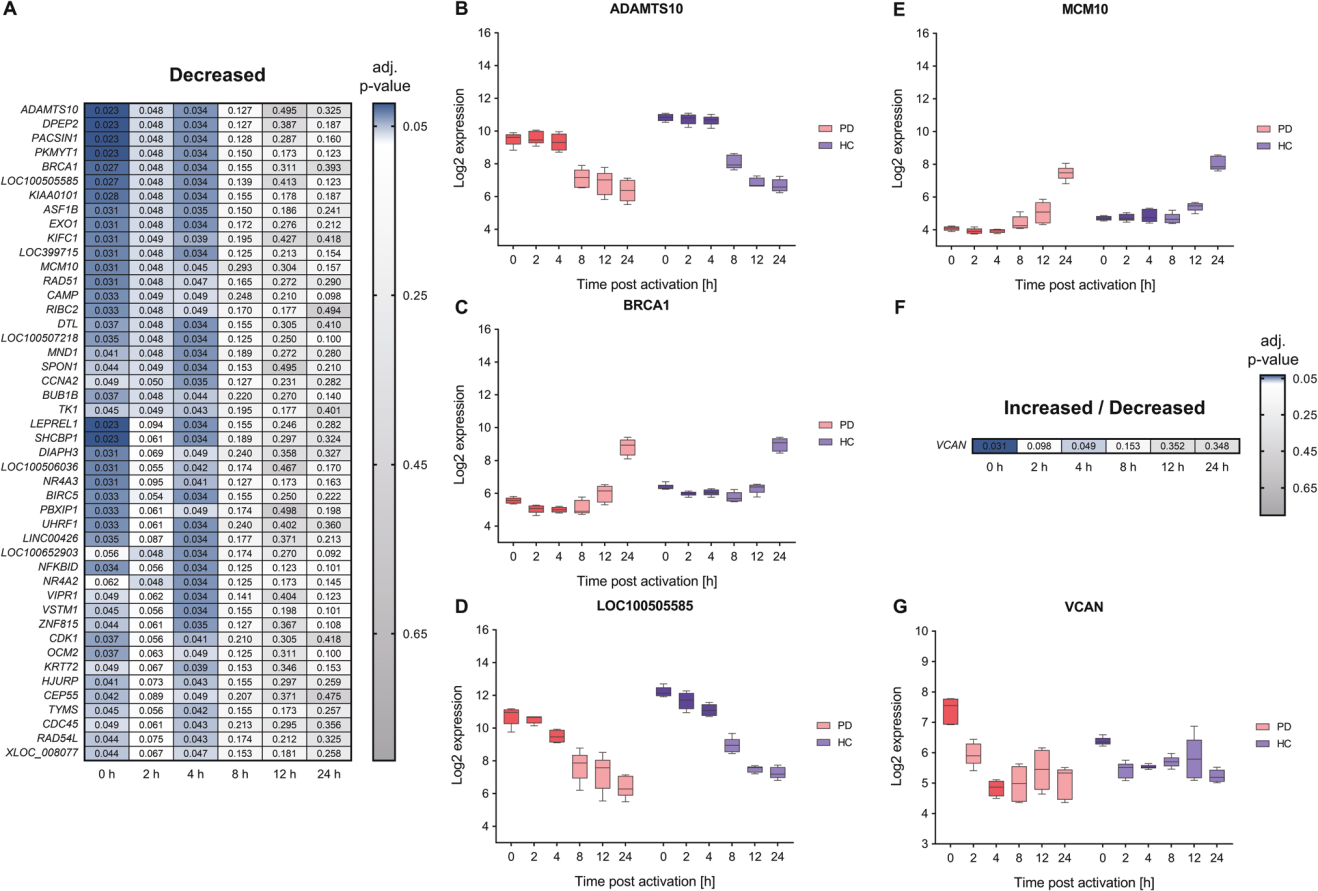
Comparative analysis of time-course transcriptomics data show decreases of T cell gene expression in PD. **A**, **F** A total 46 core deregulated genes with an adjusted p-value ≤0.05 and a median log_2_ FC ≥0.5 or ≤−0.5 for the comparison between PD and HC groups showed a significant RNA increase at more than one time-point of the T cell activation course. One core gene (*VCAN1*) showed significant RNA alternation during the T cell activation time-course. Corresponding FDR adjusted *p*-values of the analyzed time-points (0, 2, 4, 8, 12, and 24 h) are depicted for the comparison between PD and HC groups. **B**–**E**, **G** Exemplary log2 time-course expression data of ADAMTS10, BRCA1, LOC100505585, MCM10, and of VCAN1 are shown for PD and HC groups. The boxes show the range from the 25th to 75th percentiles with the median indicated by horizontal lines within the boxes and the total expression ranges per by whiskers. Significant differences between PD and HC at the time points are indicated by darker colors.

There were 46 of the core deregulated genes with a decreased RNA expression at least at two time-points in PD samples during the T cell activation time-course (Fig. 5A). These encode among others the extracellular protease ADAMTS10, the nuclear phosphoprotein BRCA1, the uncharacterized gene LOC100505585 and the replication initiation factor MCM10 (Fig. 5B–E). All corresponding RNAs showed significant expressional deregulation during 0–4 h of T cell activation. Median log_2_ fold changes of the comparison between PD and HC samples within this timeframe were in the range of −1.23 to −1.39 for ADAMTS10 and of −0.73 to −1.06 for BRCA1. Corresponding FDR adjusted p-values were in the range of 2.29 × 10^−2^–3.40 × 10^−2^ for both genes. Log_2_ fold changes of LOC100505585 and MCM10 were in the range −1.05 to −1.62 and −0.61 to −0.76, respectively. FDR adjusted p-values were in the range of 2.29 × 10^−2^–3.40 × 10^−2^ and of 3.13 × 10^−2^–4.83 × 10^−2^, respectively.

A single mRNA (extracellular matrix regulator VCAN1) revealed an alternation of increased RNA expression at 0 h (with a log_2_ fold change of 1.20 and an adj. *p*-value of 3.13 × 10^−2^) and a decreased RNA expression at 4 h (with a log_2_ fold change of −0.66 and an adj. p-value of 4.86 × 10^−2^) in PD samples (Fig. 5F, G).

Notably and as summarized in Supplementary table 2, the above-mentioned deregulated genes can be related to PD, neurodegeneration, and autoimmune diseases and are currently being under investigation for therapeutic use.

### Specification of deregulated miRNA expression in context with PD

**Fig. 6:**
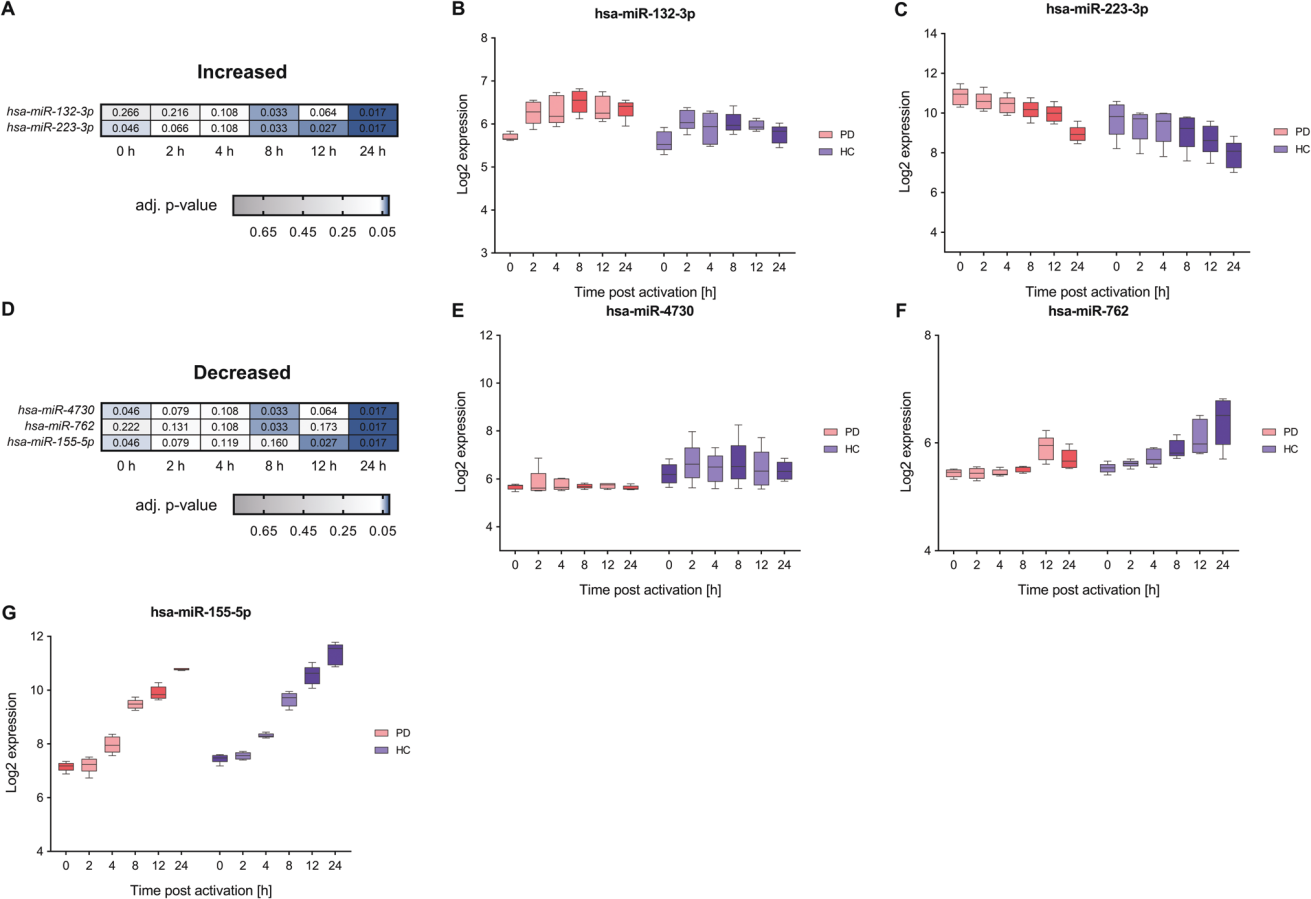
Comparative analysis of time-course expression data show deregulation of microRNA expression in PD. **A**, **D** Two core deregulated miRNAs with an adjusted *p*-value ≤ 0.05 and a median log_2_ FC ≥0.5 or ≤−0.5 for the comparison between PD and HC groups showed a significantly increased expression and three a significantly decreased expression at more than one time-point of the T cell activation course. Corresponding FDR adjusted p-values of the analyzed time-points (0, 2, 4, 8, 12, and 24 h) are depicted for the comparison between PD and HC groups. **B**, **C** and **E**–**G** Exemplary log2 time-course data are shown for hsa-miR-132-3p, hsa-miR-223-3p with an increased expression in PD and **E**–**G** for hsa-miR-4730, hsa-miR-762, hsa-miR-155-5p with a decreased expression in PD. The boxes show the range from the 25th to 75th percentiles with the median indicated by horizontal lines within the boxes and the total expression ranges per by whiskers. Significant differences between PD and HC at the time points are indicated by darker colors.

We next performed time-resolved expression analyses of the miRNome. In contrast to the transcriptome analyses we identified only very few miRNAs, according to the above applied criteria (i.e., time-course expression changes and deregulation in PD patients with significant differences at more than one time-point) (Fig. 6).

Hsa-miR-132-3p showed significantly increased expression at 8 h and at 24 h in PD samples. The according median log_2_ fold changes between PD and HC samples were 0.59 and 0.58, corresponding FDR adjusted *p*-values were 1.66 × 10^−2^ and 3.26 × 10^−2^, respectively. Hsa-miR-223-3p showed significantly increased expression at 0 h and between 8 h–24 h in PD samples. Log_2_ fold changes were in a range between 0.94 and 1.38. Corresponding FDR adjusted p-values were in the range between 1.66 × 10^−2^–4.63 × 10^−2^.

Hsa-miR-4730 showed significantly decreased expression in PD samples at 0 h, 8 h and 24 h, hsa-miR-762 at 8 h and 24 h, and hsa-miR-155–5p at 0 h, 12 h and 24 h. The according log_2_ fold changes of the comparison between PD and HC samples were in the overall range of −0.46 to −0.83 (hsa-miR-4730), −0.26 to −0.84 (hsa-miR-762) and −0.30 to −0.80 (hsa-miR-155-5p). Corresponding FDR adjusted p-values ranged from 1.66 × 10^−2^–4.63 × 10^−2^, 1.66 × 10^−2^–3.26 × 10^−2^ and 1.71 × 10^−2^–4.63 × 10^−2^, respectively.

MiRNAs usually exert rather moderate effects as frequently shown by decreased mRNA levels of their respective target genes [22, 23]. Putative targets of the above-described miRNAs were identified by inverse correlations of the according miRNA and mRNA time-course changes. For each time-point, median log_2_ FCs were determined for the comparison between PD and HC groups. An inverse Pearson´s correlation coefficient (PCC) ≤−0.5 was chosen for the matching of resulting miRNA and mRNA time-course changes. As an additional criterion, a median log_2_ FC decrease level of at least −0.3 was chosen for putative targets of the miRNAs that showed an increased expression in context with PD. A median log_2_ FC of ≥0.3 was chosen for putative targets of miRNAs with a decreased expression in PD. Corresponding analyses for hsa-miR-132-3p and hsa-miR-223-3p identified 1,525 and 862 target genes, respectively. Analyses for hsa-miR-4730, hsa-miR-155-5p and hsa-miR-762 identified 1,293, 1,747 and 1,101 genes, respectively, which are subject to direct or secondary miRNA regulatory effects. To identify direct miRNA targets we included information on strong experimental target validation as determined by miRTargetLink 2.0 [24] (Supplementary table 3). Amongst others, we identified *CRK* as target of miR-132-3p [25] with a link to T cell migration [26], and *TP53INP1* as target of miR-155-5p [27–33] with a link to cellular stress response [34].

### Determination of deregulated signaling pathways in CD4+ T cells of PD patients

**Fig. 7:**
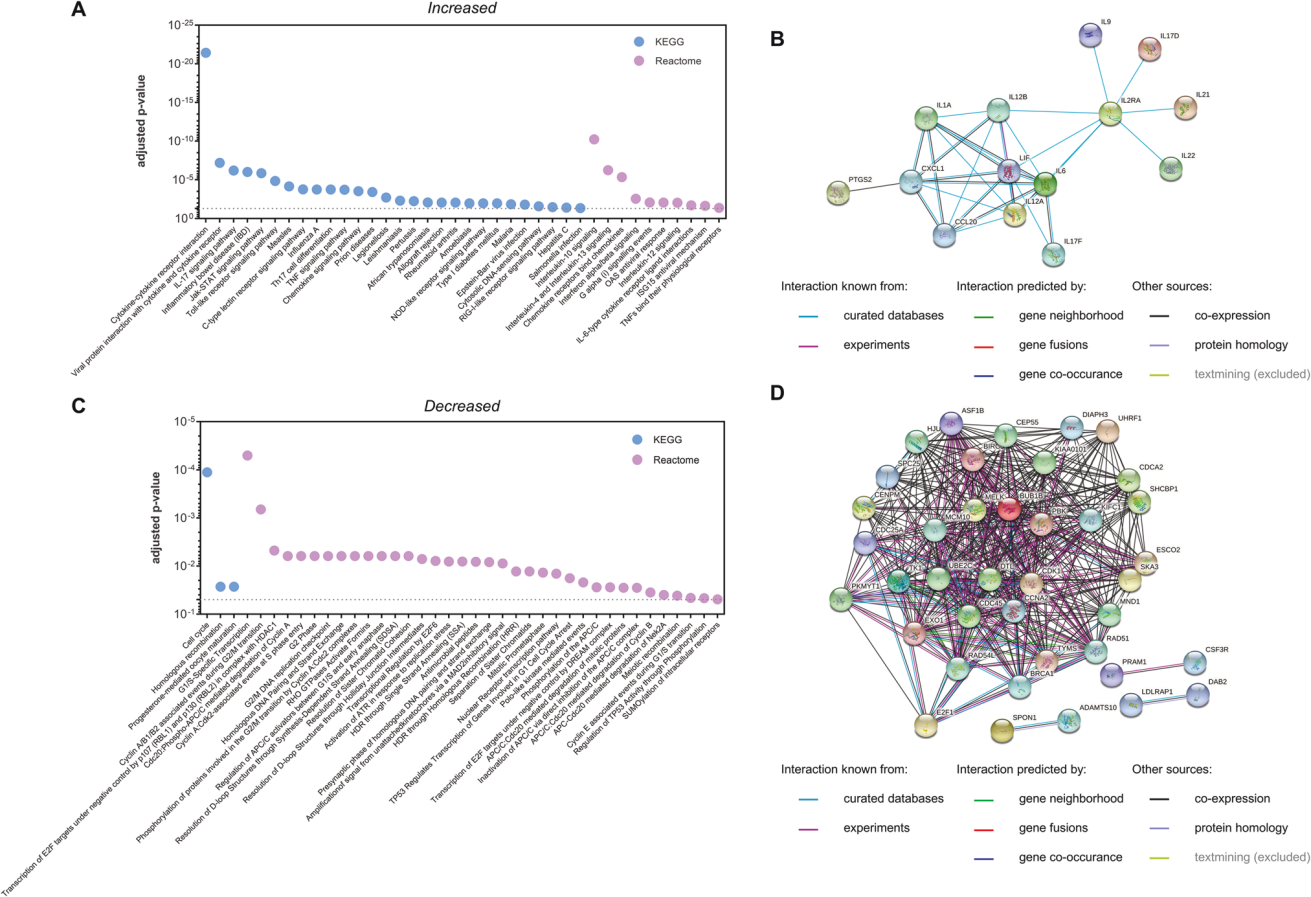
Enrichment analysis of differentially expressed genes identifies deregulated signaling pathways in CD4+ T cells of PD patients. Over-representation analysis (ORA) and protein interaction network analysis of core deregulated genes in PD. **A**, **C** Corresponding FDR adjusted *p*-values are shown for the ORA results of “KEGG” and “Reactome” databases. Protein interaction networks generated for genes with increased (**B**) and decreased (**D**) expression were exported from STRING database (v 11.5).

To identify functional consequences of the T cell expressional deregulation, we performed pathway analyses for the 172 core deregulated genes. We separately analyzed the 67 genes with increased RNA expression for pathways with increased activity in PD and the 89 genes with decreased RNA expression for pathways with decreased activity in PD. We excluded 16 genes that did not show a clear direction of the RNA deregulation from the further analysis. Gene set enrichment analyses i.e., over-representation analyses (ORAs) were conducted by the in silico tool “Genetrail 3.2” [35]. Using “KEGG” (“Kyoto Encyclopedia of Genes and Genomes” [36]) and “Reactome” [37] databases, ORA analyses uncovered a total of *n* = 38 significantly enriched categories for the genes with increased RNA expression in PD samples. These categories included “IL-17 signaling pathway” (adj. *p*-value 6.13 × 10^−7^), “Jak-STAT signaling pathway” (adj. *p*-value 1.33 × 10^−6^), “TNF signaling pathway” (adj. p-value 1.84 × 10^−4^), “Interleukin-10 signaling” (adj. *p*-value 5.58 × 10^−11^), “G alpha (i) signaling events” (adj. *p*-value 8.10 × 10^−3^), and categories of autoimmune related diseases like “Rheumatoid arthritis” (adj. *p*-value 8.49 × 10^−3^) and “Type I diabetes mellitus” (adj. *p*-value 1.07 × 10^−2^). As for the genes with decreased RNA expression in PD samples, ORA analyses identifed 39 significantly enriched categories including “Cell cycle” (adj. *p*-value 1.11 × 10^−4^), “G1/S-Specific Transcription” (adj. *p*-value 5.50 × 10^−5^), “Resolution of Sister Chromatid Cohesion” (adj. *p*-value 6.15 × 10^−3^), and “Resolution of D-loop Structures through Synthesis-Dependent Strand Annealing (SDSA)” (adj. *p*-value 6.15 × 10^−3^). Corresponding results are summarized in Fig. 7A, B.

Multiple interactions of the corresponding protein products were determined by a respective protein-interaction network of increased and decreased transcripts using the STRING database (v 11.5) [38] (Fig. 7B and D).

### Increase of IL-17 signaling, Th17 and IL-17 producing gamma delta T cells in PD

**Fig. 8:**
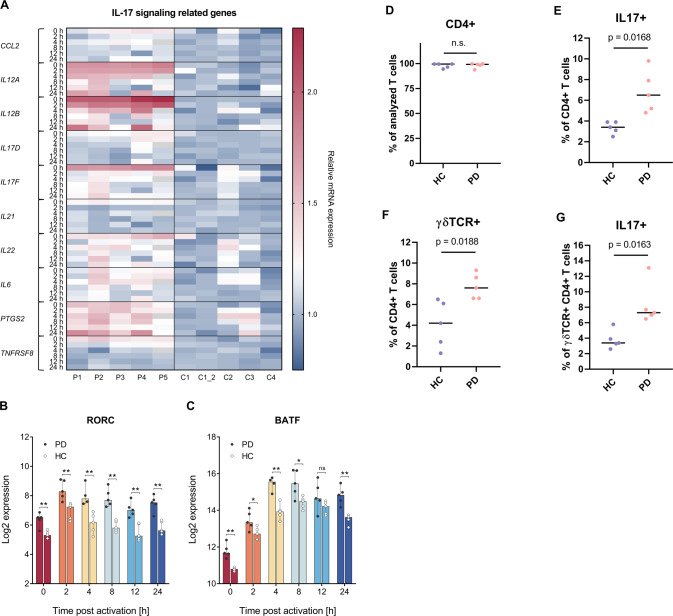
Time-course transcriptomics data and T cell subtype composition highlight deregulation of IL-17 signaling and related changes in IL-17 producing T cell types in PD. **A** Core genes with an increased expression in PD samples indicate deregulation of IL-17 signaling. Corresponding expression data for PD patients (P1, P2, P3, P4, P5) are shown in relative comparison to HCs (C1, C1_2, C2, C3, C4). The data were normalized for each of the represented genes to the median result of HCs at the respective time-point. **B**, **C** Time-course mRNA expression data (0, 2, 4, 8, 12, and 24 h) of the transcription factors RORC and BATF are shown for PD patients and HCs. Median results are indicated by bars and total expression ranges by single data points. *P*-values for the comparison between PD and HC groups are indicated for the individual time-points by asterisks (ns: not significant; *p-value ≤ 0.05; **p-value ≤ 0.01). **D**–**G** Flow cytometric analysis was done for CD4+ T cells, 6 h after CD4+ T cell activation. **D** Purity of the isolated CD4+ T cells was confirmed for HC and PD groups. **E**, **F** Significant increase of Th17 (IL-17+) and gamma delta (γδTCR+) subtypes were detected amongst the CD4+ T cells of the PD group. **G** Significant increase of IL-17 producing T cells was detected amongst the γδTCR + CD4+ T cells subpopulation (γδTCR + IL-17+) of the PD group. Median results of HC and PD groups are indicated by horizontal lines and total percentage ranges by individual data points. *P*-values for the comparison between PD and HC groups are specified.

Numerous of the genes that we found with an increased RNA expression pattern in connection with PD, could be assigned to the KEGG category “IL-17 signaling pathway” (as already mentioned above). Representative genes such as *CCL20*, *IL12A*, *IL12B*, *IL17D*, *IL17F*, *IL21*, *IL22*, *IL6*, *PTGS2* and *TNFRSR8* showed distinct mRNA deregulation during the time-course (Fig. 8A). RORC and BATF are considered as crucial transcription factors for the induction of IL-17 signaling and Th17 development [39, 40]. Examination of the corresponding time-course expression data indicated increased mRNA levels of these key transcription factors as part of the T cell activation process (Fig. 8B, C).

These findings prompted us to perform a more detailed analysis of the cell composition for the PD samples in comparison to HCs. Flow cytometric analyses were conducted based on cryo-conserved aliquots of the CD4+ T cells that were isolated for the time-course RNA expression analyses. The median purity of the isolated CD4+ T cells was at 99.5% (range: 94.5–99.6%) and 99.3% (range: 93.8–99.7%) in HC and PD samples, respectively. T cell activation was induced for 6 h prior to specific antibody staining and FACS detection.

In line with the results from our time-course RNA expression data, the determination of peripheral CD4+ T cell subtype composition verified an increased prevalence of Th17 cells (IL-17+) within PD samples (HC: Median 3.4%, range: 2.5–3.9%; PD: Median 6.5%, range: 4.8–9.8%; *p* = 0.0168) (Fig. 8E). Additionally, we found a significant increase of gamma delta T cells (γδTCR+ ; HC: Median 4.2%, range: 1.3–6.5%; PD: Median 7.6%, range: 6.6–9.3%; *p* = 0.0188) in PD samples. Among the γδ T cells, we found a significant increase of a specific subtype i.e., IL-17 producing gamma delta T cells (γδTCR + IL-17+; HC: Median 3.4%, range: 2.6–5.8%; PD: Median 7.3%, range: 6.5–13.1%; *p* = 0.0163; Fig. 8F, G). No significant differences were detected for Th1 cells (IFNγ+; HC: Median 38.7%; PD: Median 33.4%; *p* = 0.8446), Th2 cells (IL-4+; HC: Median 6.4%; PD: Median 7.5%; *p* = 0.2012) or Treg cells (CD127^low^CD25^high^; HC: Median 0.2%; PD: Median 0.2%; *p* = 0.7606) (Supplementary Fig. 2).

## Discussion

Despite first evidence for an involvement of peripheral T lymphocytes in PD pathogenesis and their potential utility for novel therapeutic strategies, there is still a lack of studies that specify relevant deregulation of T cell pathways in context with PD [8, 15]. To decipher deregulated T cell signaling in PD, we analyzed time-resolved RNA expression patterns (0–24 h) following the in vitro activation of peripheral CD4+ T cells.

When comparing the T cell activation time-course data of our PD patients to HCs, we identified an up-regulation for miR-223-3p. An increased prevalence of this miRNA has also been detected within the blood sera of PD patients [41] and a connection to brain autoimmune diseases has several times been reported [42, 43].

Additionally, we found miR-155-5p with a significant down-regulation in the CD4+ T cell time-course data of PD patients. This miRNA has, however, formerly been reported to have an increased prevalence in the blood cells of PD mouse models [44]. Our findings of a significantly reduced miR-155-5p expression were mainly attributable to the PD patients 1–3. It is legitimate to speculate that the detected effect may be related to treatment with levodopa [45], which was given to the patients 1–3. Admittingly, it is highly challenging to pinpoint drug related effects in studies with a small number of patients that received different medical treatments. We do, however, feel that the overall RNA data in our study does not reflect the treatment schemas: While we found RNA deregulations common to all patients, each patient was treated by different drugs and drug combinations.

We also identified significant deregulation of miR-762. This miRNA has been described as a regulator of energy metabolic functions and the production of reactive oxygen species (ROS) in mitochondria [46]. Elevated production of ROS is a well-known reason for the emergence of DNA damages and is commonly associated with pathological changes in cell physiology [47]. Our finding of a miR-762 deregulation may be indicative for the according scenarios in CD4+ T cells of PD patients. We also observed deregulations of specific mRNAs, including BRCA1, MAOA, MCM10, or RAD51, which encode mitochondrial proteins and enzymes involved in maintaining the genomic integrity [48–50]. Additionally, our enrichment analysis of the transcripts that were decreased in PD identified functional links to synthesis-dependent strand annealing (SDSA) that is part of the DNA double-strand break repair pathway [51]. Our findings indicate that mitochondrial dysfunctions and a malfunction in DNA repair mechanisms likely contribute to T cell related PD pathogenesis. This assumption was further supported by analyses of a specific sub-group of alpha-synuclein specific memory T cells in PD [52]. Common genomic mutations, contributing to the development of PD, also relate to changes of mitochondrial functions and DNA repair mechanisms [53–55]. Deregulations of these processes are considered as central hallmarks of PD in neuronal cells [56]. However, the functional effects of genomic mutations may possibly not be restricted to neurons but may also have a bearing for pathological changes in other cell types, including the CD4+ T cells.

Further RNA expressional deregulation can be assigned to central immune and cytokine pathways including the IL-10, Jak-STAT and G alpha (i) signaling pathways. Shifts on corresponding pathways are commonly associated with pathological T cell functions and immune disorders [57–59]. As already mentioned in the results section, the deregulation of representative transcripts such as AREG, CCL18, HCAR3, and TNFRSF8, further supports the contribution of T cell autoimmune features to PD pathogenesis. The IL-17 signaling pathway in particular appears to play a prominent role for the development of brain related autoimmune diseases [60]. Notably, animal models also provided evidence for a link between circulating IL-17 and cognitive impairments [61]. In our analyses we found strong evidence for the deregulation of IL-17 signaling. In detail, the subtype composition of corresponding peripheral CD4+ T cell samples revealed a higher incidence of cells that were assigned to the Th17 subtype. In line with our results, Sommer et al. showed an increased prevalence of IL-17-producing T cells in patients at early stages of PD [62]. Interleukin-1 (IL1) that is secreted by activated microglia in context with PD pathogenesis [63] is a critical cytokine for Th17 cell differentiation [64, 65]. As shown for other neurodegenerative diseases [66] a crosstalk between the brain and periphery may provide a potential explanation for our findings of increased TH17 counts in PD peripheral blood samples. In addition, we found an increased prevalence of a specific subtype of IL-17 producing gamma delta T cells in PD patients. To our knowledge, the involvement of this cell type in PD pathogenesis has not been reported yet. IL-17 producing gamma delta T cells can be activated, independently of any T cell receptor stimulation, through cytokines such as IL1 [67] and are considered to play an important role for autoimmune diseases by amplifying Th17 responses [68–70]. It is conceivable that this cell type contributes to the development of an autoimmune reaction as part of the PD pathogenesis.

In summary, our time-resolved RNA expression analyses of in vitro activated CD4+ T cells uncovered comprehensive RNA expressional deregulation in PD. Associated cellular processes point to a mitochondrial dysfunction and a disruption of DNA repair in CD4+ T cells of PD patients. Various connections to autoimmunity and to central PD features are highlighted. We found strong indications for the pathomechanistic relevance of IL-17 signaling and provide first evidence for the involvement of IL-17 producing γδ-T cells in PD. Our analyses refer to a relatively small number of exemplary cases. Extended analyses with larger patient groups will help to assess their potential for future clinical implications.

## Materials and Methods

### PD patients and healthy controls

Epidemiological studies show an increased prevalence of PD in connection with progressing age and with the male sex [1, 71, 72]. Based on this, the PD patients (*n* = 5) of this pilot study constitute a cohort of elderly male (age 53–85 yrs.; non-smokers), representative for different stages of disease development by the Hoehn and Yahr Scale [73, 74]. Corresponding HCs (*n* = 4) were matched for age (age 53–63 yrs.; non-smokers) and gender. One HC was carried out twice (C1 and C1_2), representing independent replicates from the same healthy donor but from two different days of blood collection (at a time distance of seven months). Basic subject data, including age, stage of disease, reported PD onset, clinical features, comorbidities and medications, are summarized in Supplementary table 1. Peripheral blood samples for subsequent T cell isolation (27 ml per donor) were drawn to lithium heparin containing collection tubes (S-Monovette, Sarstedt AG & Co. KG, Numbrecht, Germany). Written informed consents were obtained from all subjects (ethics approval ID: Ethical vote No. 213/08) and a previous immune reaction was excluded to the time-point of blood collection by the analysis of total blood counts from an additional aliquot (2 ml).

### CD4+ T cell isolation and collection of activated time-course samples

CD4+ T cells were isolated from peripheral blood samples by density gradient centrifugation, followed by negative selection with the Human CD4+ T cell Isolation Kit (Miltenyi Biotech, Bergisch Gladbach, Germany). The isolated cells were suspended with RPMI 1640 medium (Life Technologies GmbH, Darmstadt, Germany; with 10% v/v heat inactivated fetal bovine serum (Biochrom GmbH, Berlin, Germany) and 1% v/v penicillin-streptomycin (100 U/ml)) and incubated in 25 mm flasks overnight. At the following day, the cells were seeded in a 96 well format (350,000 cells/well) and were in vitro activated by the human T cell activation/expansion kit (αCD2/αCD3/αCD28 MACSiBead particles, Miltenyi Biotec GmbH, Bergisch Gladbach, Germany). Cellular samples were collected from different wells at 0, 2, 4, 8, 12 and 24 h after activation for subsequent RNA extraction as formerly described [17]. Time-course collection resulted in a total of *n* = 30 samples from PD patients (*n* = 5) and controls (*n* = 4 + *n* = 1 (C1 replicate)), respectively.

### Flow cytometry and analysis of CD4+ T cell subtype composition

Isolated CD4+ T cells from PD patients and healthy controls were stimulated with phorbol-12-myristate-13-acetate (PMA; 5 ng/ml)/ionomycin (500 ng/ml) (both from Sigma-Aldrich, Taufkirchen, Germany) for 6 h. After 2 h, brefeldin A (10 mg/ml; Sigma) was added. Cells were fixed using BD Bioscience Cytofix/Cytoperm Kit and stained using anti-CD4 (clone RPA-T4, AB_395752), anti-IFNγ (clone 4 S.B3, AB_2738952), anti-IL-4 (clone REA895, AB_2726799), anti-IL-17 (clone CZ8-23G1, AB_2752081), anti-CD127 (clone HIL-7R-M21, AB_2744279), anti-CD25 (clone M-A251, AB_2744336) and anti-γδTCR (clone 11F2, AB_2733698) and analyzed by flow cytometry (FACS CantoII, BD Biosciences). For statistical comparisons, of the FACS results and for the analysis of CD4+ T cell subtype specific transcription factor (RORC and BATF) mRNA expression data, unpaired t tests were performed assuming a normal distribution of the data.

### RNA extraction and determination of time-resolved expression profiles

Cellular RNA was extracted, quality controlled, quantified and analyzed by microarray systems from Agilent Technologies (One-Color, Human SurePrint G3) as detailed in a previous publication [17]. For the time-resolved transcriptome analysis one sample (patient P5) yielded no data at the 4 h time-point.

### Data processing

For both miRNA and transcriptome microarrays, raw expression values were processed using the limma R-package [75]. A background correction (method = normexp, offset = 16), quantile normalization, and log2 transformation were conducted. For the transcriptome data, a batch correction (‘removeBatchEffect’ method of the limma R-package) was applied to account for potential variations, due to different microarray batches.

### Statistical analysis

For the statistical analysis of the expression values, we used a two-step approach. In the first step, we identified genes or miRNAs that showed a change in expression during the analyzed 24 h period after T cell activation. For these genes, we then compared expression differences between PD patents and HCs in the second step.

To determine the expression change of a given miRNA or gene in the analyzed time frame, we calculated their maximum deviation from the initial time point using the log2 fold-change for each sample individually. We then aggregated the scores for the PD and the HC groups, respectively, using the median. Finally, we select all genes or miRNAs that appeared with an absolute fold-change of at least 1.5 in one on the two analyzed groups.

To compare gene expression differences between PD patients and HCs, we calculated log_2_ fold-changes between the median values in both groups and conducted a shrinkage t-test [76]. For all analyses, the p-values were FDR adjusted [77].

### Enrichment analysis

All enrichment analyses in this study were performed using version 3.2 of the GeneTrail web service [35]. For the analysis of deregulated biological processes we applied over-representation analyses for each test set using all protein coding genes as a reference set. All resulting p-values were FDR adjusted [78].

### Protein network analyses

Protein interaction networks were generated using the STRING database (v 11.5) [38], excluding text-mining from the selection of active interaction sources and hiding disconnected nodes within the networks.

## Supplementary information

Supplementary material

## Data Availability

The datasets of the current study are available on the GEO database repository. The super-series number of the study is #GSE202667. Accession numbers for time-resolved transcriptome profiles and time-resolved miRNA profiles are #GSE202665 and #GSE202666, respectively.
