# Supplementary material for: Time-resolved RNA signatures of CD4+ T cells in Parkinson’s disease
Source: Cell Death Discov. 2023 Jan 21;9:18. doi: 10.1038/s41420-023-01333-0 (PMC9867723; doi:10.1038/s41420-023-01333-0)
Supplement: Supplementary file 1 — Supplementary material [file 41420_2023_1333_MOESM1_ESM.pdf]

Supplementary

1. Supplementary figures:

Supplementary figure 1

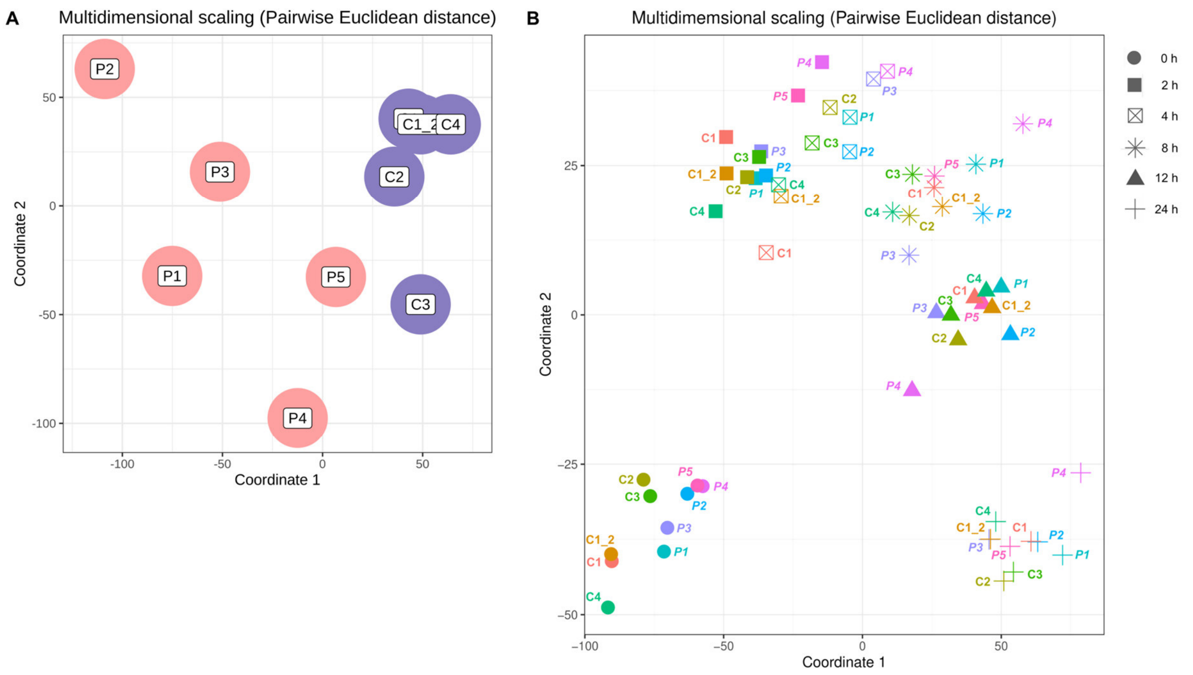

Supplementary figure 1: Multidimensional scaling highlights the separation between healthy controls and PD samples based on time-course transcriptomics data.

For the multidimensional scaling plots of the overall time-resolved transcriptomics data, pair-wise euclidean distance was determined for all of the analyzed samples (**A**) and for the different time-points (**B**), respectively.

Supplementary figure 2

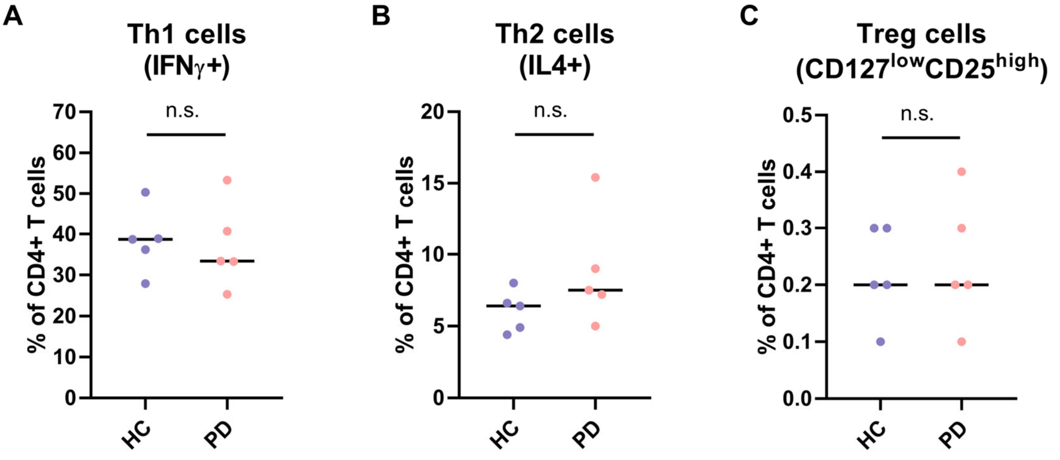

Supplementary figure 2: Percentage proportions of Th1, Th2 and Treg T cell subtypes amongst the peripheral CD4+ T cell of PD patients and HCs.

Flow cytometric analysis was done for CD4+ T cells, 6 h after CD4+T cell activation. (**A**) Th1 (IFNγ+), (**B**) Th2 (IL4+) and (**C**) Treg ($CD127^{low}CD25^{high}$) T cell subtype proportions showed no significant differences between HC and PD groups. Median results of HC and PD groups are indicated by horizontal lines and total percentage ranges by individual data points.

2. Supplementary tables:

Supplementary table 1: Basic data of PD patients and healthy controls (HCs).

#	Description	Age	Gender	Stage of disease (according Hoehn & Yahr Scale)	Patient reported onset of PD	Clinical PD features	PD medication	Comorbidities	Co-medication
**P1**	PD patient	75	Male	4	more than 15 years ago	Akinetic rigid subtype	Levodopa preparation (Levodopa - Benserazide), Levodopa preparation (Levodopa - Carbidopa - Entacapone), Ropinirole, Rivastigmine	Osteochondrosis, Anaemia, Heart failure NYHA III, Triple-vessel Coronary artery disease, Chronic renal insufficiency III°, Gastroesophageal reflux, Esophagitis	Pantoprazole 20mg/d Acetylic salicylic acid 100mg/d Torasemide 10mg/d Eplerenone 25mg/d atorvastatine 40mg/d Ezetimib 10mg/d
**P2**	PD patient	85	Male	4	Six years ago	Intermediate subtype, patient suffers of	Levodopa preparation (Levodopa - Benserazide),	none	Clopidogrel 75 mg/d

						strong fluctuation of PD medication efficacy	Levodopa preparation (Levodopa - Cabidopa – Entacapone)		
**P3**	PD patient	76	Male	2.5	more than 15 years ago	Akinetic rigid subtype, beginning cognitive impairment	Levodopa preparation (Levodopa - Benserazide), Levodopa preparation (Levodopa - Carbidopa - Entacapone), Apomorphine, Rivastigmine	Aortic valve insufficiency (mild, I°), mitral valve insufficiency (mild, I°), Hypertension, Right bundle branch block, Restless-legs-syndrome, Cataract	Fampridine 10mg/d doxepine 25mg/d ramipril 10mg/d pantoprazole 20mg/d atorvastatine 10mg/d
**P4**	PD patient	53	Male	1	one year ago	Intermediate subtype	Pramipexole	None	none
**P5**	PD patient	57	Male	1	Recently (this year)	Akinetic rigid subtype	Rasagiline, Ropinirole	Type B Gastritis (H. pylori-associated), Hypertension	Nebivolole 5mg/d ramipril 10mg/d
**C1**	Healthy Control (HC)	63	Male	0 (Healthy)					

**C1_2**	Healthy Control (HC) (replicate)	63	Male	0 (Healthy)					
**C2**	Healthy Control (HC)	69	Male	0 (Healthy)					
**C3**	Healthy Control (HC)	66	Male	0 (Healthy)					
**C4**	Healthy Control (HC)	53	Male	0 (Healthy)					

Supplementary table 2: Function of genes with differential RNA expression levels in T cells of PD.

Function of genes that were significantly deregulated between PD and HCs at more than one time-point of the T cell activation course. Emphasis is given to cellular functions, former associations with PD, neurodegeneration and immune diseases, and therapeutic approaches.

Gene	Direction of transcriptional deregulation in CD4+ T cells of PD patients	Reported gene function	Refs. (literature)
CCL18	Increased	C-C motif chemokine ligand; chemoattraction of immune cells (e.g. lymphocytes); potential link to PD (plasma, glucocerebrosidase variants)	[1], [2]
AREG	Increased	Growth-factor; type 2 cytokine; regulation of immune functions and inflammation; potential link to PD (knock-out mouse models); potential link to autoimmunity; currently on focus as potential target for innovative drugs (other pathologies)	[3], [4], [5], [6], [7]
MAOA	Increased	Monoamine oxidase; mitochondrial enzyme; regulation of T cell immunity; additional impact on neurotransmission; potential link to PD (upregulated by α-synuclein); currently on focus as potential target for innovative drugs (other pathologies)	[8], [9]
HCAR3	Increased	Hydroxycarboxylic acid receptor; impact on G protein signaling; modulator of cellular metabolism and immune responses; potential link to PD (upstream of monocyte deregulation); currently on focus as potential target for innovative drugs (other pathologies)	[10], [11]
ADAMTS10	Decreased	ADAMTS (a disintegrin and metalloproteinase domain with thrombospondin type-1 motifs) family member; extracellular protease, regulation of cell-cell communication; with impact on inflammatory responses; potential link to PD (gene variants)	[12], [13], [14]
BRCA1	Decreased	Nuclear phosphoprotein; role for transcriptional regulation and DNA repair; modulation of T cell activation; potential link to PD (mitochondrial dysfunction); currently on focus as potential target for innovative drugs (other pathologies)	[15], [16], [17]
LOC100505585	Decreased	Uncharacterized; potential link to abnormal psychomotor behavior (peripheral blood leukocytes)	[18]
MCM10	Decreased	Replication initiation factor; protection from DNA damage; telomere maintenance; relevant for canonical immune function	[19], [20], [21]

TNFRSF8	Increased	Membrane-associated TNF receptor; potential link to autoimmunity; currently on focus as potential target for innovative drugs (central nervous system diseases)	[22], [23], [24]
VCAN	Increased/Decreased	Extracellular matrix regulator; regulation of immune functions and inflammation; potential link to PD (deregulation in substancia nigra)	[25], [26]

References of Supplementary table 2:

1. Schutyser E, Richmond A, Van Damme J. Involvement of CC chemokine ligand 18 (CCL18) in normal and pathological processes. *J Leukoc Biol* 2005, **78**(1)**:** 14-26.

2. Galper J, Balwani M, Fahn S, Waters C, Krohn L, Gan-Or Z*, et al.* Cytokines and Gaucher Biomarkers in Glucocerebrosidase Carriers with and Without Parkinson Disease. *Mov Disord* 2021, **36**(6)**:** 1451-1455.

3. Zaiss DMW, Gause WC, Osborne LC, Artis D. Emerging functions of amphiregulin in orchestrating immunity, inflammation, and tissue repair. *Immunity* 2015, **42**(2)**:** 216-226.

4. Akundi RS, Huang Z, Eason J, Pandya JD, Zhi L, Cass WA*, et al.* Increased mitochondrial calcium sensitivity and abnormal expression of innate immunity genes precede dopaminergic defects in Pink1-deficient mice. *PLoS One* 2011, **6**(1)**:** e16038.

5. Sisto M, Lisi S, Lofrumento DD, Ingravallo G, Mitolo V, D'Amore M. Expression of pro-inflammatory TACE-TNF-alpha-amphiregulin axis in Sjogren's syndrome salivary glands. *Histochem Cell Biol* 2010, **134**(4)**:** 345-353.

6. Zaiss DM, van Loosdregt J, Gorlani A, Bekker CP, Grone A, Sibilia M*, et al.* Amphiregulin enhances regulatory T cell-suppressive function via the epidermal growth factor receptor. *Immunity* 2013, **38**(2)**:** 275-284.

7. Singh SS, Chauhan SB, Kumar A, Kumar S, Engwerda CR, Sundar S*, et al.* Amphiregulin in cellular physiology, health, and disease: Potential use as a biomarker and therapeutic target. *J Cell Physiol* 2022, **237**(2)**:** 1143-1156.

8. Wang X, Li B, Kim YJ, Wang YC, Li Z, Yu J*, et al.* Targeting monoamine oxidase A for T cell-based cancer immunotherapy. *Sci Immunol* 2021, **6**(59).

9. Jia C, Cheng C, Li T, Chen X, Yang Y, Liu X*, et al.* alpha-Synuclein Up-regulates Monoamine Oxidase A Expression and Activity via Trans-Acting Transcription Factor 1. *Front Aging Neurosci* 2021, **13:** 653379.

10. Kapolka NJ, Isom DG. HCAR3: an underexplored metabolite sensor. *Nat Rev Drug Discov* 2020, **19**(11)**:** 745.

11. Wnorowski A, Wnorowska S, Kurzepa J, Parada-Turska J. Alterations in Kynurenine and NAD(+) Salvage Pathways during the Successful Treatment of Inflammatory Bowel Disease Suggest HCAR3 and NNMT as Potential Drug Targets. *Int J Mol Sci* 2021, **22**(24).

12. Zhou M, Lin Y, Lu L, Zhang Z, Guo W, Peng G*, et al.* Association of ADAM10 gene variants with sporadic Parkinson's disease in Chinese Han population. *J Gene Med* 2021, **23**(3)**:** e3319.

13. Redondo-Garcia S, Peris-Torres C, Caracuel-Peramos R, Rodriguez-Manzaneque JC. ADAMTS proteases and the tumor immune microenvironment: Lessons from substrates and pathologies. *Matrix Biol Plus* 2021, **9:** 100054.

14. Cain SA, Mularczyk EJ, Singh M, Massam-Wu T, Kielty CM. ADAMTS-10 and -6 differentially regulate cell-cell junctions and focal adhesions. *Sci Rep* 2016, **6:** 35956.

15. Lu L, Huang H, Zhou J, Ma W, Mackay S, Wang Z. BRCA1 mRNA expression modifies the effect of T cell activation score on patient survival in breast cancer. *BMC Cancer* 2019, **19**(1)**:** 387.

16. Takaoka M, Miki Y. BRCA1 gene: function and deficiency. *Int J Clin Oncol* 2018, **23**(1)**:** 36-44.

17. Miyahara K, Takano N, Yamada Y, Kazama H, Tokuhisa M, Hino H*, et al.* BRCA1 degradation in response to mitochondrial damage in breast cancer cells. *Sci Rep* 2021, **11**(1)**:** 8735.

18. Zhang Y, You X, Li S, Long Q, Zhu Y, Teng Z*, et al.* Peripheral Blood Leukocyte RNA-Seq Identifies a Set of Genes Related to Abnormal Psychomotor Behavior Characteristics in Patients with Schizophrenia. *Med Sci Monit* 2020, **26:** e922426.

19. Chattopadhyay S, Bielinsky AK. Human Mcm10 regulates the catalytic subunit of DNA polymerase-alpha and prevents DNA damage during replication. *Mol Biol Cell* 2007, **18**(10)**:** 4085-4095.

20. Thu YM, Bielinsky AK. MCM10: one tool for all-Integrity, maintenance and damage control. *Semin Cell Dev Biol* 2014, **30:** 121-130.

21. Baxley RM, Leung W, Schmit MM, Matson JP, Yin L, Oram MK*, et al.* Bi-allelic MCM10 variants associated with immune dysfunction and cardiomyopathy cause telomere shortening. *Nat Commun* 2021, **12**(1)**:** 1626.

22. Shinoda K, Sun X, Oyamada A, Yamada H, Muta H, Podack ER*, et al.* CD30 ligand is a new therapeutic target for central nervous system autoimmunity. *J Autoimmun* 2015, **57:** 14-23.

23. Cano-Gamez E, Soskic B, Roumeliotis TI, So E, Smyth DJ, Baldrighi M*, et al.* Single-cell transcriptomics identifies an effectorness gradient shaping the response of CD4(+) T cells to cytokines. *Nat Commun* 2020, **11**(1)**:** 1801.

24. Sun X, Yamada H, Shibata K, Muta H, Tani K, Podack ER*, et al.* CD30 ligand/CD30 plays a critical role in Th17 differentiation in mice. *J Immunol* 2010, **185**(4)**:** 2222-2230.

25. Wight TN, Kang I, Evanko SP, Harten IA, Chang MY, Pearce OMT*, et al.* Versican-A Critical Extracellular Matrix Regulator of Immunity and Inflammation. *Front Immunol* 2020, **11:** 512.

26. Downs M, Sethi MK, Raghunathan R, Layne MD, Zaia J. Matrisome changes in Parkinson's disease. *Anal Bioanal Chem* 2022, **414**(9)**:** 3005-3015.

Supplementary table 3: Putative direct mRNA targets of the PD deregulated microRNAs.

For each time-point, median log2 FCs of the mRNA data are denoted for the comparison between PD and HC groups (Median FC PD vs. Control). Pearson´s correlation coefficients (PCC≤-0.5) are depicted for the matching of corresponding miRNA time-course changes. A median log2 FC decrease level of at least -0.3 (highlighted in green) was assumed for putative targets of the miRNAs that yielded an increased expression in context with PD. A median log_2 FC increase level of at least 0.3 (highlighted in blue) was assumed for putative targets of the miRNAs that yielded a decreased expression in context with PD. Literature on experimental target validation is referenced.

miRNA	PD vs. Control	Likely target	PCC (Median FC, time-course RNA data)	Median FC PD vs. Control (0 h)	Median FC PD vs. Control (2 h)	Median FC PD vs. Control (4 h)	Median FC PD vs. Control (8 h)	Median FC PD vs. Control (12 h)	Median FC PD vs. Control (24 h)	Literature on experimental target validation
hsa-miR-132-3p	Increased	CRK	-0.708	-0.221	-0.064	-0.310	-0.576	-0.028	-0.417	1
hsa-miR-132-3p	Increased	CDKN1A	-0.679	0.559	0.189	0.627	-0.216	-0.473	-0.189	2
hsa-miR-132-3p	Increased	TJAP1	-0.542	-0.235	-0.081	-0.230	-0.282	-0.081	-0.324	1
hsa-miR-223-3p	Increased	PRDM1	-0.679	0.113	0.580	0.496	0.004	-0.324	-0.006	3
hsa-miR-223-3p	Increased	LIF	-0.672	0.624	0.692	0.847	0.240	-0.368	0.252	4
hsa-miR-223-3p	Increased	CCL3	-0.561	0.156	-0.101	-0.107	-0.439	-1.024	-0.363	5
hsa-miR-155-5p	Decreased	TP53INP 1	-0.900	0.354	0.279	0.379	0.271	0.621	0.466	6, 7, 8, 9, 10, 11, 12
hsa-miR-155-5p	Decreased	LDOC1	-0.819	0.014	-0.271	-0.099	0.118	0.471	0.338	13
hsa-miR-155-5p	Decreased	CYP2U1	-0.808	-0.076	-0.120	-0.312	0.010	0.323	0.189	8, 14
hsa-miR-155-5p	Decreased	HOMEZ	-0.800	0.096	0.073	0.086	-0.093	0.432	0.145	15
hsa-miR-155-5p	Decreased	ANAPC1 6	-0.782	0.025	-0.029	0.132	0.206	0.334	0.308	8
hsa-miR-155-5p	Decreased	C16orf62	-0.752	0.162	0.260	0.092	0.210	0.293	0.387	8
hsa-miR-155-5p	Decreased	ZNF652	-0.751	-0.072	0.069	-0.335	0.038	0.783	0.189	8, 16, 17
hsa-miR-155-5p	Decreased	ETS1	-0.748	-0.167	0.239	-0.031	0.052	0.348	0.286	8, 17, 18, 19
hsa-miR-155-5p	Decreased	FAM199X	-0.651	-0.025	0.001	0.043	0.229	0.306	0.203	8
hsa-miR-155-5p	Decreased	FGF7	-0.629	0.144	0.232	0.025	0.097	0.443	0.153	20
hsa-miR-155-5p	Decreased	MYB	-0.606	-0.346	0.343	0.183	0.101	0.379	0.411	21, 22

hsa-miR-155-5p	Decreased	C3orf18	-0.559	0.283	0.171	0.460	0.203	0.294	0.608	8, 23
hsa-miR-155-5p	Decreased	HIVEP2	-0.511	-0.693	-0.589	0.017	0.115	-0.015	0.322	8, 16, 24
hsa-miR-155-5p	Decreased	RPTOR	-0.504	-0.029	0.008	-0.166	0.064	-0.035	0.379	25

References of Supplementary table 3:

1. Cambronne XA, Shen R, Auer PL, Goodman RH. Capturing microRNA targets using an RNA-induced silencing complex (RISC)-trap approach. *Proc Natl Acad Sci U S A* 2012, **109**(50)**:** 20473-20478.

2. Wu S, Huang S, Ding J, Zhao Y, Liang L, Liu T*, et al.* Multiple microRNAs modulate p21Cip1/Waf1 expression by directly targeting its 3' untranslated region. *Oncogene* 2010, **29**(15)**:** 2302-2308.

3. Liang L, Nong L, Zhang S, Zhao J, Ti H, Dong Y*, et al.* The downregulation of PRDM1/Blimp-1 is associated with aberrant expression of miR-223 in extranodal NK/T-cell lymphoma, nasal type. *J Exp Clin Cancer Res* 2014, **33**(1)**:** 7.

4. Sun G, Li H, Wu X, Covarrubias M, Scherer L, Meinking K*, et al.* Interplay between HIV-1 infection and host microRNAs. *Nucleic Acids Res* 2012, **40**(5)**:** 2181-2196.

5. Dorhoi A, Iannaccone M, Farinacci M, Fae KC, Schreiber J, Moura-Alves P*, et al.* MicroRNA-223 controls susceptibility to tuberculosis by regulating lung neutrophil recruitment. *J Clin Invest* 2013, **123**(11)**:** 4836-4848.

6. Gironella M, Seux M, Xie MJ, Cano C, Tomasini R, Gommeaux J*, et al.* Tumor protein 53-induced nuclear protein 1 expression is repressed by miR-155, and its restoration inhibits pancreatic tumor development. *Proc Natl Acad Sci U S A* 2007, **104**(41)**:** 16170-16175.

7. Wang Y, Scheiber MN, Neumann C, Calin GA, Zhou D. MicroRNA regulation of ionizing radiation-induced premature senescence. *Int J Radiat Oncol Biol Phys* 2011, **81**(3)**:** 839-848.

8. Xu G, Fewell C, Taylor C, Deng N, Hedges D, Wang X*, et al.* Transcriptome and targetome analysis in MIR155 expressing cells using RNA-seq. *RNA* 2010, **16**(8)**:** 1610-1622.

9. Saito Y, Suzuki H, Tsugawa H, Imaeda H, Matsuzaki J, Hirata K*, et al.* Overexpression of miR-142-5p and miR-155 in gastric mucosa-associated lymphoid tissue (MALT) lymphoma resistant to Helicobacter pylori eradication. *PLoS One* 2012, **7**(11)**:** e47396.

10. Zhang CM, Zhao J, Deng HY. MiR-155 promotes proliferation of human breast cancer MCF-7 cells through targeting tumor protein 53-induced nuclear protein 1. *J Biomed Sci* 2013, **20**(1)**:** 79.

11. Zhang C, Zhao J, Deng H. 17beta-estradiol up-regulates miR-155 expression and reduces TP53INP1 expression in MCF-7 breast cancer cells. *Mol Cell Biochem* 2013, **379**(1-2)**:** 201-211.

12. Liu F, Kong X, Lv L, Gao J. MiR-155 targets TP53INP1 to regulate liver cancer stem cell acquisition and self-renewal. *FEBS Lett* 2015, **589**(4)**:** 500-506.

13. Skalsky RL, Samols MA, Plaisance KB, Boss IW, Riva A, Lopez MC*, et al.* Kaposi's sarcoma-associated herpesvirus encodes an ortholog of miR-155. *J Virol* 2007, **81**(23)**:** 12836-12845.

14. Diener C, Hart M, Kehl T, Rheinheimer S, Ludwig N, Krammes L*, et al.* Quantitative and time-resolved miRNA pattern of early human T cell activation. *Nucleic Acids Res* 2020, **48**(18)**:** 10164-10183.

15. Slezak-Prochazka I, Kluiver J, de Jong D, Smigielska-Czepiel K, Kortman G, Winkle M*, et al.* Inhibition of the miR-155 target NIAM phenocopies the growth promoting effect of miR-155 in B-cell lymphoma. *Oncotarget* 2016, **7**(3)**:** 2391-2400.

16. Yin Q, McBride J, Fewell C, Lacey M, Wang X, Lin Z*, et al.* MicroRNA-155 is an Epstein-Barr virus-induced gene that modulates Epstein-Barr virus-regulated gene expression pathways. *J Virol* 2008, **82**(11)**:** 5295-5306.

17. Neilsen PM, Noll JE, Mattiske S, Bracken CP, Gregory PA, Schulz RB*, et al.* Mutant p53 drives invasion in breast tumors through up-regulation of miR-155. *Oncogene* 2013, **32**(24)**:** 2992-3000.

18. Romania P, Lulli V, Pelosi E, Biffoni M, Peschle C, Marziali G. MicroRNA 155 modulates megakaryopoiesis at progenitor and precursor level by targeting Ets-1 and Meis1 transcription factors. *Br J Haematol* 2008, **143**(4)**:** 570-580.

19. Zhu N, Zhang D, Chen S, Liu X, Lin L, Huang X*, et al.* Endothelial enriched microRNAs regulate angiotensin II-induced endothelial inflammation and migration. *Atherosclerosis* 2011, **215**(2)**:** 286-293.

20. Pottier N, Maurin T, Chevalier B, Puissegur MP, Lebrigand K, Robbe-Sermesant K*, et al.* Identification of keratinocyte growth factor as a target of microRNA-155 in lung fibroblasts: implication in epithelial-mesenchymal interactions. *PLoS One* 2009, **4**(8)**:** e6718.

21. Imig J, Motsch N, Zhu JY, Barth S, Okoniewski M, Reineke T*, et al.* microRNA profiling in Epstein-Barr virus-associated B-cell lymphoma. *Nucleic Acids Res* 2011, **39**(5)**:** 1880-1893.

22. Muylkens B, Coupeau D, Dambrine G, Trapp S, Rasschaert D. Marek's disease virus microRNA designated Mdv1-pre-miR-M4 targets both cellular and viral genes. *Arch Virol* 2010, **155**(11)**:** 1823-1837.

23. Louafi F, Martinez-Nunez RT, Sanchez-Elsner T. MicroRNA-155 targets SMAD2 and modulates the response of macrophages to transforming growth factor-beta. *J Biol Chem* 2010, **285**(53)**:** 41328-41336.

24. Yin Q, Wang X, Fewell C, Cameron J, Zhu H, Baddoo M*, et al.* MicroRNA miR-155 inhibits bone morphogenetic protein (BMP) signaling and BMP-mediated Epstein-Barr virus reactivation. *J Virol* 2010, **84**(13)**:** 6318-6327.

25. Tsuchiya M, Kalurupalle S, Kumar P, Ghoshal S, Zhang Y, Lehrmann E*, et al.* RPTOR, a novel target of miR-155, elicits a fibrotic phenotype of cystic fibrosis lung epithelium by upregulating CTGF. *RNA Biol* 2016, **13**(9)**:** 837-847.
